# The Length and Content of General Practice Consultation in Two Urban Districts of Beijing: A Preliminary Observation Study

**DOI:** 10.1371/journal.pone.0135121

**Published:** 2015-08-10

**Authors:** Guanghui Jin, Yali Zhao, Chao Chen, Wenji Wang, Juan Du, Xiaoqin Lu

**Affiliations:** 1 Department of General Practice, School of General Practice and Continuing Education, Capital Medical University, Beijing, P. R. China; 2 Puren Hospital, Beijing, P. R. China; Centre for Health and Population Sciences, Hull York Medical Center, UNITED KINGDOM

## Abstract

**Background:**

Community health service center (CHSC) and community health service station (CHSS) are the main institutions where general practitioners (GPs) deliver primary care in the urban area of China. Motivated by incentive policies, visits to community health service institutions (CHSIs) increased gradually in recent years, but concerns had been raised on the quality of general practice consultation. This is a preliminary study aimed to investigate the existing problems of general practice consultation in Beijing and provide practical evidence for developing relevant policies.

**Methods:**

Six GPs from 2 CHSCs and 3 CHSSs were selected by purposive sampling. The GPs were observed for 4 or 5 consecutive days during January 2013 to March 2013. The length and content of consultations were recorded in structured observation forms. Quantitative description was applied to describe the median, percentage and frequency of variables.

**Results:**

A total of 1135 consultations were observed. The most frequent reason for consultations was specific prescription (61.6%), followed by presenting symptoms (20.7%), check-up (9.1%), counseling (5.4%), transfusion & injection (3.0%) and sickness certificate (0.2%). The median consultation length of all consultations was 2.0 minutes. The GPs prescribed in 81.0% of the consultations, on the other hand, history taking, physical examination, explanation of illness and health education only took place in 27.0%, 28.0%, 21.9% and 17.7% of the consultations respectively.

**Conclusions:**

The adequacy of consultation length in CHSIs is in doubt. Most patients visited the CHSIs for prescription renewal. Health promotion e.g. health education are not adequately provided in consultations. The quality of general practice consultations was jeopardized by the large amount of patient flow for medicine renewal. Policies should be adjusted to reduce unnecessary consultations. Further studies are in need to evaluate the outcome and influencing factors of general practice consultation in China.

## Introduction

Plagued by the inefficiency and inequality of health care system after economic reforms in 1978, the State Council of China decided to initiate health care reform in 1997 and indicated that one focus of the reform was on developing community health service (CHS) to revive its once prosperous primary care system [1]. Following policies released in 1999 further defined CHS’s role as the foundational component of primary care system [2]. More sophisticated policies regarding finance, human resource, pharmaceutical price, health insurance regulation, etc in CHS were issued by the Ministry of Health and other national level departments in 2006 [3], thereafter, a nationwide CHS network was gradually established, till the end of 2012, 8,182 CHCs and 25,380 CHSs had been set up in China[4]. CHSIs comprise CHSCs and CHSSs, which are expected to provide medical care, preventive care, health promotion, rehabilitation, health education and family planning for the citizens. A CHC usually has department of general practice clinic, traditional Chinese medicine clinic, resuscitation room, immunization, women health, children health, laboratory tests, ultra sound, ECG, pharmacy etc [5]. There should be at least 6 GPs and 9 nurses in a CHC. Besides, a community health center should have at least 1000 m^2^ of space [5].The CHSSs are smaller affiliated institutions of CHSCs and intended to improve geographic access, covering areas further away from CHSCs. Its space should be at least 150 m^2^. A CHSS usually contains departments of general practice clinic, preventive care and pharmacy. There should be at least 2 GPs and 1 nurse in a CHSS [5]. General practitioner is the key member of CHS team delivering primary care for the community.

In the urban area, three major policies were implemented to facilitate the utilization of CHS: (1) expanding medical insurance coverage to achieve universal coverage and till 2011 only 5.2% population have no medical insurance in China [4]; (2) CHSIs have a higher reimbursement proportion than secondary or tertiary hospitals for outpatient visits, e.g. the reimbursement proportion is 90% for CHSIs and 70% for hospitals in Beijing [6]; (3) the prescription medicines were dispensed at the procurement price without markups [7]. Supported by these incentive policies, visits to CHSIs increased from 122 million to 599 million by nearly 4 fold between 2005 and 2012 in China [4, 8, 9]. Nevertheless, concerns were raised that most of the increased visits were chronic patients coming for prescription renewal, patient still prefer to go to see specialists in a hospital instead of consulting the general practitioners when they get ill [10]. Preventive care including health education and health promotion were poorly delivered in general practice consultations [11]. Brief consultation with low quality in general practice was once a concern in the UK, and time availability is considered as an important aspect for assessing consultation quality [12]. A systematic review indicated consultation length could affect consultation contents, e.g. more health promotion will be delivered in longer consultation and better patient enablement and satisfaction outcome will be achieved [13].

China’s new blueprint for health care reform in 2009 reinforced CHS’s future role as the first contact of health care system [14]. However, few studies were found to explore the quality general practice consultation in China. This study aimed to investigate the length and content of general practice consultations in CHSIs of Beijing and existing problems, and provide practical evidence for developing policies regarding promoting the quality general practice consultation in the urban area of Beijing.

## Methods

### Ethics statement

The study was approved by the Ethical Committee of the Capital Medical University, Beijing, China. Written informed consent was obtained from each participating GP in this study. Written informed consents from the patients were not required by the ethical committee because these data were collected for subsequent analysis of the characteristics of the consultations, and acquisition of written consent could potentially interfere the consultation process. So Patients were informed about the study in the waiting room before seeing the GPs and their verbal consent were obtained, the date and investigator’s name were recorded in a verbal consent form. The only patient data collected were the main reason for consultation, consultation length and content, no individually identifying patient characteristics were collected nor reported. All participants' information was kept confidential and will only be tracked with coding number anonymously.

### Participants

Purposive sampling was applied in the study for selection of GPs. It is a type of non-probability yet efficient and valid sampling method, and setting up appropriate criteria of selection is important for selecting proper informants and saving time and effort [15]. To ensure the representativeness of selected GPs and a stable and adequate amount of visits for the GPs in clinics, general practice experts and researchers were consulted before sampling to define the criteria of selection. Two CHSCs in 2 urban districts of Beijing and 3 affiliated CHSSs were selected considering the following criteria: (1) ensured accessibility and availability for the patients; (2) stable amounts of visits; (3) available for the study. The selection criteria of GPs included: (1) rank above attending doctor; (2) worked in the community for over 5 years; (3) mainly worked in the clinic; (4) available for the study. Administrative leaders of the CHSIs who are responsible for quality control, performance evaluation, research and teaching were asked to give advices for selecting GPs met the criteria. Six general practitioners were selected, 3 of whom were selected from 2 CHSCs and the other 3 from 3 CHSSs respectively.

### Data collection

Participating GPs were visited by postgraduate students while providing ambulatory care on 4 or 5 consecutive days during January 2013 to March 2013. All the postgraduate students were full-time master candidates in general practice and were trained before the investigation. The consultation sample consisted of consecutive patients seen by the GPs during the observation. To avoid biasing their behavior, no specific hypotheses e.g. ‘general practice consultation is short’ was shared with the patients and GPs, they were only informed that this was a study of the length and content of general practice consultation. The observers were seated in the least intrusive corner of the consultation room and will not talk to the GPs and patients. The observers collected data on main reasons for consultation, consultation length and content by direct observation in consultation rooms. Watch was used to record consultation length, and a self-designed observation form was adopted to record patients’ main reasons for the consultation and the content of consultation. Besides seeing patients, many GPs also need to undertake public health and administrative work in CHSIs which could influence the consultations, so these work were also timed and recorded in the form. The form was developed with reference to literatures and social study researchers were consulted to revise the form. Eighty consultations for a GP during 2 days were observed as trial study and revisions were made to the observation form.

### Analysis

Descriptive statistics (median, frequencies and percentages) were used to describe the demographic information of GPs, reasons for consultations, consultation length and content. Analyses were conducted using Statistical Package for Social Science (SPSS) for Microsoft Windows (Version 17.0, SPSS Inc, Chicago, IL, USA).

## Results

### Basic information of the GPs

Table 1 showed the demographic information of 6 GPs participating in this study. All the 6 GPs were female, 3 of whom worked in CHSCs, and the other 3 worked in CHSSs. Four GPs were observed for 4 consecutive days, 2 GPs were observed for 5 consecutive days. Total 1135 consultations for 6 GPs were observed. The largest amount of consultations per day was 91.0 for a chief doctor in CHSC, the fewest consultations per day was 11.0 for an attending doctor in CHSS. Four of the GPs also undertook a lot of public health (e.g. screening) and administrative (e.g. reporting information, organizing the pharmacy) tasks during observation and the workload was measured by minutes.

**Table 1:** Basic information of participating GPs.

GP	Gender	Age	Institution	Title	Days	Consultations	Per day	Public health workload(min)	Administrative workload (min)
1	Female	42	CHSC	Chief doctor	4	364	91.0	0	0
2	Female	37	CHSC	Attending doctor	4	266	66.5	0	0
3	Female	40	CHSC	Associate chief doctor	4	155	38.8	234	40
4	Female	41	CHSS	Attending doctor	5	95	19.0	548	321
5	Female	43	CHSS	Associate chief doctor	4	200	50.0	186	304
6	Female	36	CHSS	Attending doctor	5	55	11.0	495	427

Abbreviation: GP: general practitioner, CHSC: community health service center, CHSS: community health service station.

### Reasons for the consultations

Reasons for the 1135 consultations were divided into 6 types. Specific prescription accounted for the most of consultations (699, 61.6%), followed by consultations presenting with one or more symptoms (235, 20.7%), check-up (103, 9.1%), counseling (61, 5.4%), transfusion & injection (34, 3.0%) and sickness certificate (3, 0.3%). The proportion of different reasons for the consultations by institutions was shown in Table 2.

**Table 2:** Different reasons for the consultations by institutions (n = 1135).

Reason for the consultations	No. of consultations (Percentage)		
	CHSCs	CHSSs	All
Specific prescription	499 (63.5%)	200 (57.2%)	699 (61.6%)
Presenting with symptoms	196 (25.0%)	39 (11.1%)	235 (20.7%)
Check-up	29 (3.7%)	74 (21.1%)	103 (9.1%)
Counseling	33 (4.2%)	28 (8.0%)	61 (5.4%)
Transfusion & injection	25 (3.2%)	9 (2.6%)	34 (3.0%)
Sickness certificate	3 (0.4%)	0	3 (0.2%)
Total	785 (100.0%)	350 (100.0%)	1135 (100.0%)

Abbreviation: GP: general practitioner, CHSC: community health service center, CHSS: community health service station

### Consultation length

Consultation length was described by different consultation reasons. The median consultation length of all the visits was 2.0 minutes. The median consultation length of patients presenting with symptoms was 3.0 minutes, followed by specific prescription (2.0 minutes), check-up (2.0 minutes), counseling (2.0 minutes) and sickness certificate (1.0 minutes). 86.1% of all the consultations were in the range of “1~” minutes. 73.6% of consultations presenting with symptoms were in the range of “1~” minutes, 18.7% were in the range of “6~” minutes. Most consultations of specific prescription (89.7%), check-up (89.3%), transfusion and injection (76.5%) and counseling (91.8%) were in the range of “1~” minutes. All visits for sickness certificate were in the range of “1~” minutes, see Table 3.

**Table 3:** Distribution of consultations in different ranges of consultation length.

Reasons for the consultations	Number of consultations(percentage)				
	1 ~ (min)	6~ (min)	11~ (min)	16~ (min)	Total
Presenting with symptoms	173 (73.6%)	44 (18.7%)	16(6.8%)	2(0.9%)	235(20.7%)
Specific prescription	627 (89.7%)	63 (9.0%)	8(1.2%)	1(0.1%)	699 (61.6%)
Check-up	92 (89.3%)	8 (7.8%)	3(2.9%)	0	103 (9.1%)
Sickness certificate	3 (100%)	0	0	0	3 (0.2%)
Transfusion & injection	26 (76.5%)	8 (23.5%)	0	0	34 (3.0%)
Counseling	56 (91.8%)	4 (6.6%)	1(1.6%)	0	61 (5.4%)
Total	977 (86.1%)	127 (11.2%)	28(2.4%)	3(0.3%)	1135

### Contents of consultation

The 6 GPs prescribed for most of the patients (81.0%). Though history taking, physical examination, explanation of illness and health education only took place in 27.0%, 28.0%, 21.9% and 17.7% of all the consultations respectively. Contents of consultation by reasons for the consultations were shown in Table 4.

**Table 4:** Contents of consultation by reasons for the consultations.

Reasons for the consultations	Contents of consultation(frequency)				
	History taking	Physical examination	Explanation of illness	Prescription	Health education
Presenting with symptoms	180(76.6%)	135(57.4%)	122(51.9%)	195(83.0%)	77(32.8%)
Specific prescription	85(12.2%)	117(16.7%)	60(8.6%)	693(99.1%)	82(11.7%)
Check-up	14(13.6%)	58(56.3%)	17(16.5%)	13(12.6%)	29(28.2%)
Sickness certificate	0	0	0	0	0
Transfusion & injection	11(32.4%)	2(5.9%)	3(8.8%)	6(17.6%)	0
Counseling	17(27.9%)	6(9.8%)	46(75.4%)	12(19.7%)	13(21.3%)
Total	307 (27.0%)	318 (28.0%)	248 (21.9%)	919 (81.0%)	201 (17.7%)

## Discussion

CHS is expected to be the cornerstone of health care system in China. A nationwide network of CHS has been established in terms of number of CHSIs and visits to CHSIs also increased gradually [4]. But whether CHS is playing an effective gate-keeping role is unknown. In our study the biggest attractiveness of GP clinic for the patients seemed to be prescription renewal (61.6%), only 20.7% of the patients had a real problem to consult. And the reasons for consultations were different between CHSCs and CHSSs, CHSCs had relatively more visits presenting common problems (e.g. coughing, diarrhea), because CHSCs have more sophisticated facility and instruments than CHSSs. CHSSs are closer for residents than CHSCs, so it is more convenient to visit the CHSSs for regular check up of blood glucose, etc. and counseling. The disproportionate visits of prescription renewal can be attributed to several causes. Aging of the population, prevalence of chronic disease had brought higher health care need in community [16]. The policy incentives including the privilege of 20% more reimbursement than hospital and essential medicines dispensed at the procurement price made CHSIs more attractive, especially for patients with low income [17,18,19]. Let alone the geographical convenience as well as better availability and accessibility than hospitals. Patients with chronic diseases usually have to renew their prescriptions every 2 weeks or 1 month under the regulations of health insurance regulation institutions [20]. And majority of these patients are old patients who feel more familiar and comfortable to see GPs than specialists in the hospital [18, 21]. So, the relatively small-size CHSIs are confronted with a large amount of chronic patients who just want to refill their prescription for at least once a month. In our study, a chief doctor in CHSC saw 91.0 patients per day during the observation, the visits for GPs in CHSSs were relatively fewer because CHSSs were smaller than CHSCs. And the amount for each GP also varies, because GPs with higher title tend to see more patients. Some GPs also need to undertake a lot of public health and administrative workload, so they may see fewer patients. However, when real health problems (e.g. fluctuant blood pressure) emerge, most patients’ first choice would still be the hospital. Patients in China usually associate large and prominent hospitals with high quality and professional medical care, and prefer to visit these hospitals for all spectrums of diseases. This phenomenon would not be changed with financial incentives in CHSIs alone [10], because the competency of GPs is still skeptical for the patients and public [22]. The government publicized the advantages of CHSIs in waiting time, prescription medicine price and better accessibility and available, but quality of care had not been the focus yet [10]. CHSIs are acting as a cushion for hospital’s large clinic burdens not the gatekeeper of health care system. The development of CHS has only achieved partial success[10].

GPs have to spend most of their time prescribing for the patients renew their medicine, most patient just want their prescription and GPs will not do anything more than prescribing. Besides seeing patients in the clinic, GPs still have to undertake a lot of public health and administrative work in CHSIs, because there is still a huge gap in human resources of general practice nurses, social workers and administrative staff, etc [23]. Moreover, there is no appointment system in most CHSIs [24], All the patients can walk into the consultation room at any time, long lines of patients waiting for getting prescription is a common scene in the consultation room. So the GPs have to sacrifice consultation length and quality for efficiency. The median consultation length of all the visits was only 2.0 minutes in this study. Even for patients presented symptoms, the median consultation length was only 3.0 minutes, because the GPs sometimes have to make the consultation faster for patients with a problem to consult, when there are many patients waiting to renew their prescription. And 86.0% of all the consultations were within 6 minutes.

The role of general practice varies between different countries and health care systems, so does consultation length. A cross sectional study in 2002 investigating six European countries (including Belgium, Germany, Netherlands, Spain, Switzerland and UK) found out the overall mean consultation length in these six countries was 10.7 minutes [25]. In an observational study investigating 4,454 consecutive ambulatory visits to 138 family physicians at 84 community family practices in northeast Ohio of the US, the mean consultation length were 9.2 minutes, 12.1 minutes, 9.5 minutes respectively in rural, inner city and suburban area [26]. Few studies on general practice consultation were conducted in China, though mean consultation length of ambulatory visits was found to be 7.8 minutes in an observational study at 6 community health service institutions in 3 districts of Beijing including rural area [11]. Comparing with this study, the difference might be caused by different study design, i.e. only urban practices were included and more consultations were observed in our study. Results of our study implied the brevity of general practice consultation in urban CHSIs and consultation length of different reasons of consultations was different. Although comparison between different countries and studies might be misleading, because the contexts are different and study design varies, the adequacy of consultation length in our study is clearly in doubt.

Consultation contents had been proven to be influenced by consultation length, there would be more health promotion and less prescription in longer consultations, better outcome with regard to patient enablement and satisfaction could be attained [13]. Longer consultations could identify more psychosocial problems in patients [27] and lead to better solution [28]. In our study, only 27.0% of patients got history taking, 28.0% got physical examination, 21.9% got explanation of illness and 17.7% got health education. On the other hand, GPs prescribed for 81.0% patients of all visits, only a few preventive care or even physical examination took place. The contents of consultation were compromised for seeing more patients and more often, though seeing patients more often won’t compensate for deficiencies of longer-term properties in individual consultations [13]. It is also harmful for the GPs to maintain their competencies. An investigation in 3 cities showed the capacity of the community health facilities in providing diagnostic services was limited, and many GPs were unable to perform some essential physical examinations [29]. If they spend most of their time prescribing the medicine the patients want instead of utilizing their clinical knowledge and skills in consultations, the situation will get worse. And the patients will be more skeptical about GPs’ competencies [30].

GPs should have more power to decide the frequency of medicine renewal on the basis of patients’ stability i.e. if the patient is stable the interval for medicine renewal can be prolonged. The health insurance institutions may also adjust the policies on prescription renewal to reduce unnecessary consultations. The working conditions should be promoted in CHSIs to attract more qualified general practice nurses, social workers, administrative staff etc. to share the burden of public health and administration [31]. High prevalence of chronic diseases, more presenting problems per consultation, greater involvement in complex community based interventions, increasing patient participation with more complex interaction in modern general practice consultation require longer consultations to deal with these problems [12, 32]. It is reasonable to anticipate that an effective appointment system should be established in CHSIs, within which GPs are able to consult at a pace with balanced efficiency and quality of consultation [12]. A pilot study on appointment system in Beijing showed better outcome in patients’ overall satisfaction rate including consultation length and efficiency of consultation was also improved. Patients were arranged to visit the CHSIs during different periods of time in a day, and time spent in registration and waiting was reduced significantly [33]. Furthermore, training more qualified GPs is essential for promoting the quality of care in CHSIs and building patients’ trust in GPs’ competency [34].Till 2012, there were total 109,794 GPs in China, and only 20% of the GPs had medical education background of bachelor’s degree of medicine or beyond. The government had initiated a plan in 2011 to train 300, 000 GPs till 2020 through different pathways, including residency training program for new medical graduates and on job training programs for existing GPs, which showed the government’s determination to promote the competency and capacity of GPs [35]. Besides training more competent GPs, the government also needs to put more focus on quality improvement of general practice consultation. General practice will not be effective in gate-keeping in China, if the quality of care is not ensured for the patients [36].

## Limitations

The study adopted purposive sampling which is a non-probability but efficient sampling method. The sample was selected according to recommended criteria from general practice experts and researchers. The interpretation of findings is limited to the population under study. However, with ensured reliability and competence of the informants and representativeness of sample, data collected by purposive sampling could be as valid [15]. Using appropriate method to estimate consultation length is another issue. To time consultation length by an observer in the consultation room is one of the accurate methods to investigate the detailed components of consultation, though an observer is inevitably intrusive on the consultation [12]. So both the GPs and patients were blinded from any hypothesis of the study, they were only informed that the study was on general practice consultation length and content. The observers were seated in the least intrusive corner of the consultation room and will not talk to the GPs and patients. All the 6 participating GPs were female because the majority of GPs were female in those CHSIs selected, e.g. all GPs were female in the 3 CHSSs and the male doctors in CHSCs were reluctant to participate in the study. It could potentially influence the results, previous study indicated female GPs tend to have longer consultations for patients [26]. Patient characteristics including age, gender etc. were shown to influence consultation [37], constrained by study funding, detailed information of the patients were not collected in our study though.

This is a relatively small-scale study as the first step to investigate general practice consultation in CHSIs, and the results were mainly analyzed by statistical description. But existing defects of general practice consultation could still be clearly seen from the results. Larger scale and random sampling should be applied to evaluate the outcome and influencing factors of general practice consultation in future study.

## Conclusions

Appropriateness of consultation length in CHSIs is in doubt. Most patients visited the CHSIs for prescription renewal. Health promotion e.g. health education are not sufficiently provided during consultations. The quality of general practice consultations was jeopardized by the large amount of patient flow for medicine renewal. Policies should be adjusted to reduce unnecessary consultations. More studies are in need to evaluate the outcome and influencing factors of general practice consultation in China.
